# M1a prostate cancer: Results of a Dutch multidisciplinary consensus meeting

**DOI:** 10.1002/bco2.73

**Published:** 2021-02-03

**Authors:** Shafak Aluwini, Daniela E. Oprea‐Lager, Hilda de Barros, Niven Mehra, Herman Stoevelaar, Derya Yakar, Henk van der Poel, Dutch M1a Prostate Cancer Working Group

**Affiliations:** ^1^ Department of Radiation Oncology UMCG Groningen The Netherlands; ^2^ Department of Radiology & Nuclear Medicine Amsterdam University Medical Centers VU University Amsterdam The Netherlands; ^3^ Department of Urology Antoni van Leeuwenhoek Amsterdam The Netherlands; ^4^ Department of Medical Oncology Radboudumc Nijmegen The Netherlands; ^5^ Centre for Decision Analysis & Support Ismar Healthcare NV Lier Belgium; ^6^ Department of Radiology UMCG Groningen The Netherlands

**Keywords:** extra‐pelvic lymph node, metastatic hormone‐sensitive prostate cancer, nodal metastasis, non‐regional lymph node, PSMA‐PET/CT, recurrent prostate cancer

## Abstract

**Objectives:**

To determine the consensus of a Dutch multidisciplinary expert panel on the diagnostic evaluation and treatment of de novo and recurrent metastatic prostate cancer (PCa) limited to non‐regional lymph nodes (M1a) in daily clinical practice.

**Materials and methods:**

The panel consisted of 37 Dutch specialists from disciplines involved in the management of M1a PCa (urology, medical and radiation oncology, radiology, and nuclear medicine). We used a modified Delphi method consisting of two voting rounds and a consensus meeting (video conference). Consensus (good agreement) was defined as the situation in which ≥ 75% of the panelists chose the same option.

**Results:**

Consensus existed for 57% of the items. The panel agreed that prostate‐specific membrane antigen positron emission tomography/computed tomography (PSMA‐PET/CT) is the most appropriate standard imaging modality to identify de novo (100%) and recurrent (97%) M1a PCa. Androgen deprivation therapy (ADT) combined with radiotherapy to the prostate ± the M1a lesion(s) was most frequently considered an option for de novo M1a PCa. For M1a as recurrent disease, ADT alone, deferring treatment, or local radiotherapy to the M1a lesion(s) were judged to be the most important treatment options. However, no specific indications for treatment choice in relation to disease characteristics could be formulated.

**Conclusions:**

The Dutch consensus panel preferred PSMA‐PET/CT as the standard diagnostic modality to detect M1a PCa. Although potential treatment options were identified, explicit recommendations could not be formulated. This might (partly) be explained by the absence of high‐level clinical evidence in this subset of patients. Further research is, therefore, strongly encouraged.

## 1 INTRODUCTION

According to the TNM classification, M1a prostate cancer (PCa) is defined as the presence of non‐regional lymph nodes (LNs), that is, LNs above the bifurcation of the common iliac arteries, while other metastases (bone/visceral) are absent.^1^ The detection of M1a disease is highly dependent on the imaging modality used.^2^ Modern imaging modalities, such as positron emission tomography/computed tomography (PET/CT) and whole‐body magnetic resonance imaging (wbMRI), allow earlier and more precise identification of metastases.^2^ Between 2010 and 2018, the age‐adjusted incidence of de novo M1a PCa in The Netherlands increased from 0.47 to 1.89 cases per 100,000 population (imaging modality unspecified), and this rise may partly be explained by the use of newer, more sensitive imaging modalities during the more recent years.^3^ The median overall survival of this group of patients was 57 months.^3^ Patients with metastases limited to the non‐regional LNs have better overall survival outcomes than patients with visceral and/or bone metastases.^4^ However, evidence on treatment of M1a PCa is limited. Available prospective data on the management of de novo low‐volume metastatic disease include patients with limited (less than four) bone metastases and are, therefore, not limited to M1a disease. In addition, the diagnosis of metastatic disease was based on conventional imaging (CT and bone scan).^5^, ^6^, ^7^, ^8^, ^9^, ^10^, ^11^, ^12^, ^13^ Also in the recurrent setting, data on management of M1a disease are sparse and mostly retrospective. Few prospective studies investigated the management of oligorecurrent disease, but these were not limited to M1a disease.^14^, ^15^ Studies have shown that using newer and more sensitive imaging modalities may lead to a management change of metastatic PCa, however, the impact on oncological outcomes is unknown.^16^, ^17^ Altogether, the management of M1a PCa in daily clinical practice is surrounded by many uncertainties.^18^ Therefore, we organized a multidisciplinary consensus meeting to determine the state‐of‐the‐art on M1a disease and its clinical implications for The Netherlands.

## 2 MATERIALS AND METHODS

### 2.1 Set‐up

The consensus meeting was set up by a multidisciplinary Scientific Committee (S.A., N.M., D.O.L., H.P., and D.Y.) and an advising methodologist (H.S.). The selected approach combined the elements from the Delphi method, Nominal Group Technique, and consensus development techniques.^19^

### 2.2 Panel composition

The panel consisted of representatives from all disciplines involved in the management of M1a PCa: urology (N = 10), medical oncology (N = 7), radiation oncology (N = 7), radiology (N = 4), and nuclear medicine (N = 9). Selection of panelists was based on clinical and scientific expertise in the field of PCa, geographic spread, and availability to participate in all parts of the study.

### 2.3 Explorative survey

The first step consisted of an explorative survey on multiple statements and questions related to the definition and management of M1a PCa. The compilation of this survey was based on the clinical expertise of the Scientific Committee members and an explorative literature search for English‐language original and review articles published up to April 2020 using the National library of Medicine's PubMed database (H.B.). The search strategy included the following terms: “M1a prostate cancer,” “newly‐diagnosed M1a prostate cancer,” “de novo M1a prostate cancer,” “recurrent M1a prostate cancer,” “newly‐diagnosed low‐volume prostate cancer,” “de novo low‐volume prostate cancer,” “oligometastatic prostate cancer,” “oligorecurrent prostate cancer,” (“non‐regional lymph node” OR “distant lymph node” OR “extra‐pelvic lymph node” OR “extra‐pelvic disease” OR “nodal recurrence”) AND “prostate cancer.” The abstracts of the retrieved records were screened to identify the most relevant articles. Relevant studies mentioned in the reference list of the identified articles were also taken into account. Additionally, meeting abstracts (2019‐2020) reporting on patients with de novo or recurrent M1a disease were included.

### 2.4 Consensus process

Panelists were asked to complete the explorative survey and provide suggestions for improvement. These survey results were shared with the panelists after which a video conference took place (June 19, 2020). Based on the survey results and the panel discussion during the video conference, a second survey with revised statements and questions was compiled and sent out to the panelists 1 week after the video conference. This survey contained six statements (5‐point Likert scale for agreement), and 15 multiple‐choice questions on the following topics:

- Definition
  ○ Anatomical level for non‐regional LNs for therapeutic decisions
  ○ Attribution of inguinal and pararectal LNs to M1a disease
- Diagnosis
  ○ Indications/appropriateness of imaging (techniques) to detect/assess metastases
  ○ Next steps after suspicion of metastases on imaging
  ○ Relevance of specific imaging parameters
- Treatment de novo M1a PCa
  ○ Curative intent of treatment and indications
  ○ Endpoints for clinical studies
  ○ Potential treatments and relevant parameters for treatment choice
- Treatment M1a as recurrent disease
  ○ Potential treatments and relevant parameters for treatment choice
- All items included the option “Can't judge.”

The complete survey is shown in the supporting information S1.

### 2.5 Statistical analysis

Strong agreement (consensus) and fair agreement were defined as the situation in which ≥ 75% or 50%‐74% of the panelists, respectively, chose the same option. If the option “can't judge” was chosen, the answer was excluded from the agreement calculations.

## 3 RESULTS

Consensus existed on 57% of the items (supporting information S2).

### 3.1 Definition

The TNM classification takes the iliac bifurcation as the anatomical inferior limit for M1a^1^ and 39% of the panelists considered this also to be the most relevant level for therapeutic decision making. A similar share chose for “what is in line with the LN dissection template or irradiation field,” and a minority (22%) for the aorta bifurcation. Opinions on whether inguinal and pararectal LNs can be considered as M1a disease were highly dispersed (Table 1).

**TABLE 1:** Panel results on statements regarding the definition and diagnosis of M1a PCa

Statement				# answers (# valid answers) ^a^	Agree^b^ %	Neutral^b^ %		Disagree^b^ %	
*Definition*									
1		The following locations of lymph node metastases can be considered as M1a prostate cancer:							
		Inguinal		37 (37)	49	8		43	
		Pararectal		37 (36)	36	19		44	
*Diagnosis*									
2	If M1a is suspected on CT scan, an extra PSMA‐PET/CT scan should be performed if this may have therapeutic consequences		37 (37)		**92**		0		8
3	If a PSMA‐PET/CT scan reveals inconclusive M1a disease, a targeted MRI should still be performed for confirmation		37 (36)		28		11		61
4	In most cases, imaging is sufficient to diagnose M1a disease and anatomopathological confirmation is not required		37 (37)		**78**		5		16
5	In case of exclusive mediastinal/hilar lymph nodes, which are enlarged and show an increased uptake, it is unlikely these are metastases of prostate cancer		37 (36)		**92**		3		6
6	The presence of a supraclavicular lymph node, which shows increased uptake, may indicate a metastasis of prostate cancer, even if no other active lymph nodes are seen elsewhere		37 (35)		**89**		6		6

Note

The bold values represent statements for which ≥ 75% of the panelists chose the same option (consensus).

Abbreviations: CT = computed tomography; MRI = magnetic resonance imaging; PCa = prostate cancer; and PSMA‐PET/CT = prostate‐specific membrane antigen positron emission tomography/computed tomography.

^a^ Valid answers: “can't judge (unqualified to answer)” excluded.

^b^ Agree = categories “agree” + “strongly agree”; disagree = categories “disagree” + “strongly disagree.” % = Percentages of valid answers.

### 3.2 Diagnosis

**FIGURE 1:**
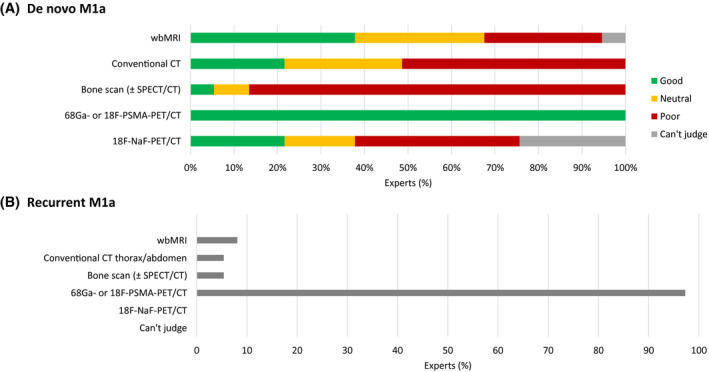
Imaging modalities to assess (or diagnose) the presence of M1a disease in (A) the de novo setting (perceived appropriateness) and (B) the recurrent setting (most recommended). ^18^F = fluorine 18; ^68^Ga = gallium 68; CT = computed tomography; Good = categories “very appropriate” + “appropriate”; NaF = sodium fluoride; PET = positron emission tomography; Poor = categories “very inappropriate” + “inappropriate”; PSMA: prostate‐specific membrane antigen; SPECT = single photon emission computed tomography; and wbMRI = whole‐body magnetic resonance imaging

**FIGURE 2:**
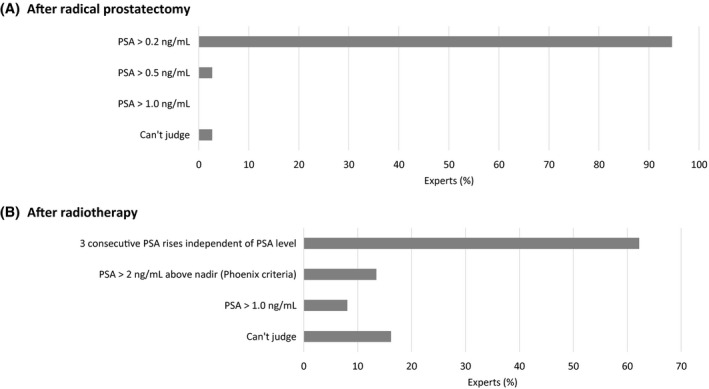
Indication for imaging (A) in case of biochemical relapse following radical prostatectomy and (B) in case of suspicion of residual disease after curative radiotherapy. PSA = prostate‐specific antigen

Indications considered most important for performing imaging for metastatic screening in PCa patients were prostate‐specific antigen (PSA) > 20 ng/mL (92%), International Society of Urological Pathology (ISUP) grade ≥ 3 (89%), and ≥ cT3 (72%). Other factors were less chosen: ≥ cT2c (36%), ISUP grade ≥ 2 (3%), and a combination of lower staging factors (17%). As standard for initial metastatic screening of PCa, ^68^Ga‐ or ^18^F‐radiolabeled‐PSMA‐PET/CT (ie, PSMA‐PET/CT) was recommended by 97% of panelists, followed by wbMRI and bone scan ± SPECT/CT (both 11%) and conventional CT (5%). More specifically, PSMA‐PET/CT was considered most appropriate for the diagnosis of M1a disease in the initial and recurrent setting by (almost) all panelists (Figure 1). There was consensus that PSMA‐PET/CT should be performed following a positive CT scan if the results would influence subsequent treatment decisions, that diagnostic imaging is generally sufficient and no pathological confirmation is needed, that the existence of exclusively suspicious mediastinal/hilar LNs is unlikely to be PCa‐related, and that increased uptake by a solitary left supraclavicular LN may indicate PCa metastasis (Table 1). Most panelists (89%) considered the combination of size, morphology, and location highly relevant when using conventional CT, while 11% found the combination of size and location to be sufficient for judging LNs. For PSMA‐PET/CT, the most relevant parameters, in addition to a higher uptake, were localization (83%), anatomical substrate on CT (78%), and size of the lesion (50%). For the recurrent setting, almost all panelists (97%) considered PSA > 0.2 ng/mL the most important indication for imaging following radical prostatectomy (RP). For biochemical recurrence after radiotherapy, there was fair agreement (74%) that three consecutive PSA rises, independent of PSA level, are most relevant in this respect (Figure 2).

### 3.3 Treatment of M1a PCa

**FIGURE 3:**
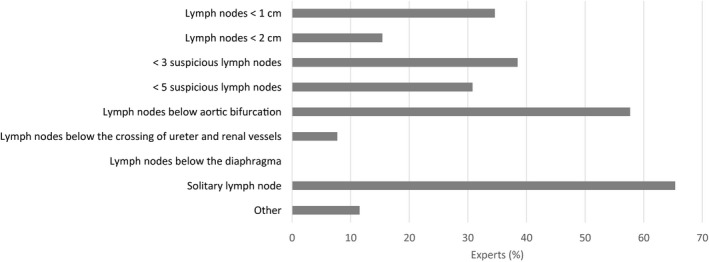
Expert opinion on situations of potentially curative M1a disease in the de novo setting. % based on the number of experts who believe a curative‐intent treatment is possible in the de novo M1a setting (N = 26/37 experts). Other included (N = 3 answers): combination of location and number of M1a lesion(s); combination of number, location, and size of M1a lesion(s) and the willingness to accept toxicity; can't judge

**FIGURE 4:**
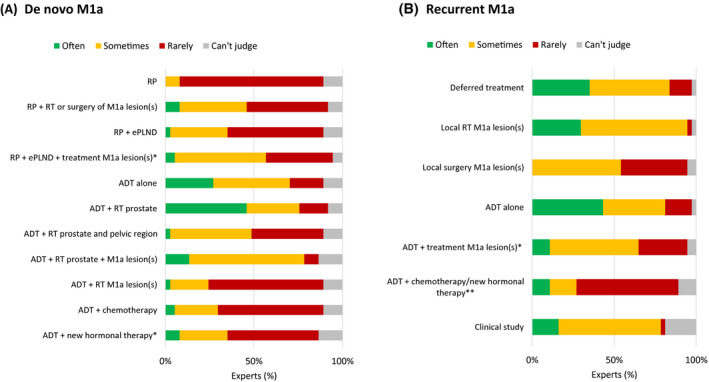
Expert opinions on eligibility of treatment options in patients with (A) de novo M1a disease and (B) recurrent M1a disease. Imaging modality not specified. Each expert needed to give his/her opinion on each treatment option. *Local RT M1a or local surgery M1a. **New hormonal therapy = abiraterone, apalutamide, or enzalutamide (androgen receptor‐targeted agents). ADT = androgen deprivation therapy; ePLND = extended pelvic lymph node dissection; RP = radical prostatectomy; and RT = radiotherapy

**FIGURE 5:**
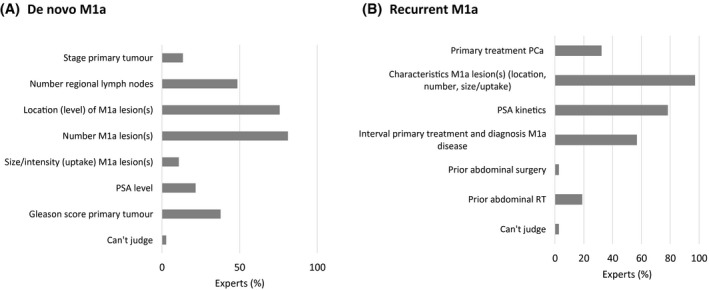
Expert opinion on most important disease‐related factors to take into account for treatment decision making in (A) de novo M1a disease and (B) recurrent M1a disease. In both the de novo and the recurrent setting, the experts were asked to indicate the three most important factors. PCa = prostate cancer; PSA = prostate‐specific antigen; and RT = radiotherapy

There was almost consensus (74%) that treatment for de novo M1a may (sometimes) have a curative intent. A solitary LN and LNs below the aorta bifurcation were most frequently mentioned as a potentially curative condition (Figure 3). Metastasis progression‐free survival was considered the most important endpoint for clinical studies into M1a PCa (53% of panelists), followed by delay of systemic treatment and overall survival (both 24%). Opinions on treatment options were diverse (Figure 4), but androgen deprivation therapy (ADT) plus radiotherapy to the prostate was most frequently considered for de novo M1a disease. A minority of panelists considered ADT plus chemotherapy (6%) or ADT plus androgen receptor‐targeted agents (ARTA) (9%) often an option for de novo patients. The most important disease‐specific factors for treatment choice in de novo M1a PCa patients included number of non‐regional LNs (83%), location of non‐regional LNs (78%), number of regional LNs (50%), and Gleason score of the primary tumor (39%) (Figure 5). For M1a as recurrent disease, ADT alone, deferring treatment or local radiotherapy of the M1a lesion(s) were chosen most frequently (Figure 4). Relevant parameters for treatment choice included characteristics of the non‐regional LNs (location, number, size, and intensity) (100%), PSA kinetics (81%), interval between primary treatment and diagnosis of M1a disease (58%) (Figure 5).

## 4 DISCUSSION

### 4.1 Definition

Although the TNM classification defines M1a as LNs outside the true pelvis (“essentially above the bifurcation of the common iliac arteries”),^1^ panelists disagreed on the most relevant anatomical level for therapeutic decisions in patients with non‐regional LNs. Dispersed opinions were also seen for considering inguinal or pararectal LNs as non‐regional node (M1a) locations. Based on TNM, inguinal and pararectal LNs should be considered as M1a and N1 disease, respectively.^1^ This is also confirmed by studies investigating the pattern of lymphatic drainage of the prostate.^20^, ^21^ Adequately defining the extent of nodal disease is critical for correct completion of registries.

### 4.2 Diagnosis

ISUP grade ≥ 3, PSA > 20 ng/mL and ≥ cT3 were considered the most common indications to perform imaging for metastatic screening. This is largely in line with the European Association of Urology (EAU) guidelines recommendations.^18^ Although the EAU guidelines do not recommend PSMA‐PET/CT for primary staging but at least abdominopelvic CT and bone scanning for metastatic screening, the panel considered PSMA‐PET/CT the most appropriate imaging modality for the initial diagnosis of metastatic disease, and also more specifically of M1a disease in both de novo and recurrent setting. This may be related to the wide experience and availability of PSMA‐PET/CT in The Netherlands and the accumulating data showing high sensitivity of PSMA‐PET/CT for LN involvement and small metastases in both primary and biochemical recurrence settings.^22^, ^23^ In addition, the recent prospective proPSMA trial showed superior diagnostic accuracy of PSMA‐PET/CT vs conventional imaging in men with high‐risk PCa before curative‐intent surgery or radiotherapy.^16^ For patients with biochemical recurrence, PSMA‐PET/CT is currently only recommended by the EAU guidelines following RP if the PSA level is > 0.2 ng/mL and if the results will influence subsequent treatment decisions, or following radiotherapy if the patient is fit for curative salvage treatment.^18^ Three consecutive PSA rises independent of the PSA level was considered to be the most common indication for imaging following curative radiotherapy, with a minority of panelists indicating PSA nadir + 2 ng/mL (Phoenix criteria) or PSA > 1.0 ng/mL. The Advanced Prostate Cancer Consensus Conference (APCCC) 2019 did not reach consensus on when to first image a patient with rising PSA following radiotherapy; nearly 60% voted for imaging prior to meeting the Phoenix criteria or voted to base the decision on additional factors besides PSA level.^24^ Indeed, data show that metastatic PCa is frequently detected with PSMA‐PET/CT in men not meeting the Phoenix criteria for biochemical recurrence following curative radiotherapy.^25^ While there was consensus that imaging is sufficient to diagnose M1a disease and pathology confirmation is not required in most cases, the APCCC 2019 panelists agreed that histopathological confirmation of PCa is needed in most patients with high suspicion of metastatic PCa.^24^

### 4.3 Treatment of M1a PCa

Opinions on treatment options for de novo M1a PCa (imaging modality unspecified) varied considerably and no consensus was obtained on a particular treatment option. Although the imaging modality for this question was unspecified, the majority of panelists may have given their opinion based on M1a disease detected on PSMA‐PET/CT as this imaging modality is widely used in clinical practice in the Netherlands. The expert opinions should be interpreted regardless of the underlying imaging modality. The variation in opinions on treatment options may (partly) be explained by the fact that currently available prospective data focus on low‐volume metastasized disease (CHAARTED definition, ie, less than four bone metastases) which differs from M1a disease.^5^, ^6^, ^7^, ^8^, ^9^, ^10^, ^11^, ^12^, ^13^ Subanalyses of M1a patients are mostly not available and if so, these lack statistical power. Additionally, these data are based on conventional imaging. During the APCCC 2019 meeting, 55% of panelists negatively answered the question if low‐volume disease defined by PET or MRI, but not evident on CT or bone scan, should be treated in the same way as low‐volume disease defined by CT and bone scan.^24^ In addition, 92% of these panelists considered it important to distinguish LN‐only disease (including non‐regional LN metastases) from disease that includes metastatic lesions at other sites.^24^ ADT plus radiotherapy to the prostate was most commonly chosen by our panel. This approach is recommended by the EAU guidelines for patients whose first presentation is low‐volume metastatic disease as defined by the CHAARTED criteria.^18^ An exploratory analysis of the STAMPEDE “M1|RT comparison” (arm H) found that the addition of prostate radiotherapy to standard of care may improve survival among men with only non‐regional LNs (M1a) or less than four bone metastases (± LNs, and no visceral metastases) regardless of location.^26^ It should be noted that the results of the STAMPEDE trial on radiotherapy to the primary cannot be extrapolated to RP. Currently there are no randomized phase III data available on the use of RP in this setting. ADT combined with chemotherapy (docetaxel) or ADT combined with ARTA was only considered by a minority of panelists. Until now, subanalyses on the use of these combinations in de novo M1a patients are not available. Although ADT alone was also chosen by the panelists as an option for de novo M1a disease, this should be considered controversial. The EAU guidelines recommend the addition of radiotherapy to the prostate (only for low‐volume disease), docetaxel, abiraterone acetate, apalutamide, or enzalutamide to ADT for patients with de novo metastatic disease who are fit enough for the regimen.^18^ Patients with de novo M1a disease were included in most of the prospective trials investigating the addition of these systemic treatments to ADT, with the exception of the TITAN trial (apalutamide), in which all patients had bone metastatic disease.^11^ Offering ADT alone to newly diagnosed metastatic patients will eventually result in progression to castration‐resistant disease and a window of opportunity would be missed. Some panelists also mentioned local treatment of the primary tumor site and M1a lesions as an option. However, metastasis‐directed therapy (MDT) of nodal disease outside the pelvis is still considered experimental.^18^ Several ongoing trials are investigating the role of MDT in addition to the treatment of the primary and standard of care systemic therapy in the de novo oligometastatic setting, including PLATON (NCT03784755) and a new arm in the STAMPEDE trial. High‐level evidence for the combination of ADT, treatment of the primary tumor and additional systemic therapy (chemotherapy or ARTA) is scarce, but this will be addressed in the ongoing PEACE‐1 trial (NCT01957436). Although clear evidence is lacking, the panelists considered number and location of M1a lesions and PSA level the three most important disease‐specific measures to decide on treatment in the de novo setting. A recent exploratory analysis of the STAMPEDE “docetaxel comparison” (arm C) showed that increased metastatic LN burden (equal to or more than five metastatic nodes) was associated with worse overall and failure‐free survival compared to patients with less than five metastatic LNs treated with ADT or ADT combined with docetaxel for metastatic hormone‐sensitive PCa.^27^ Twenty‐eight percent of the patients were diagnosed with distant LN metastasis at baseline by CT/MRI scans of which almost half had both common iliac and retroperitoneal LN metastases.

For M1a as recurrent disease, ADT alone was considered an option most frequently, followed by deferring treatment and local radiation of the M1a lesion. However, no consensus was obtained on a particular treatment option. The EAU guidelines recommend immediate systemic treatment in both asymptomatic and symptomatic metastatic patients.^18^ Deferred castration can be discussed with well‐informed asymptomatic metastatic patients provided the patient is closely monitored.^18^ In a recent retrospective study, patients with PSMA‐PET/CT para‐aortic LN metastases (M1a) following RP underwent metastasis‐directed radiotherapy (stereotactic body radiotherapy [SBRT] or conventionally fractionated external beam radiotherapy often with simultaneously integrated boost or SBRT) with or without ADT and showed a biochemical control rate of 48% at a median follow‐up of 16 months.^28^ In addition, these patients had a high progression rate (43%) outside the irradiated field. Likewise, in a small prospective single‐center study, more than two‐thirds of patients with oligorecurrent PCa, limited to the LNs in 65% of patients, developed recurrent cancer outside the area treated with SBRT after 15 months.^29^ A retrospective study showed that about one‐third of patients with biochemically recurrent disease following RP had PSMA‐avid disease that would be missed by standard nodal radiation fields.^30^ A recent systematic review showed large heterogeneities in radiotherapy regimens used for nodal oligorecurrent PCa (SBRT vs elective nodal radiotherapy) and the optimal strategy in this setting remains to be determined.^31^ Additionally, a recent retrospective study revealed that PET/CT underestimated the burden of nodal prostate recurrence.^32^ In the prospective phase II ORIOLE trial, improved progression‐free survival was seen for patients with recurrent hormone‐sensitive PCa and one to three metastases by conventional imaging who underwent MDT (stereotactic ablative radiotherapy) vs observation.^14^ Of the 54 patients included in this trial, 61% had M1a disease. Interestingly, 44% of patients treated with MDT had baseline PET‐avid lesions not included in the treatment field. Total consolidation of PET/CT‐avid lesions resulted in a lower proportion of men with progression at 6 months (5% vs 38%, *P* = .03).^14^ MDT remains controversial and one might argue that these patients might profit more from systemic therapy. Nevertheless, with the advent of PET/CT, patients at high risk of recurrence might receive improved management by early targeting of these small metastatic lesions by MDT or more aggressive multimodal strategies. However, MDT in this setting should only be offered in the context of clinical trials like the ADOPT trial (NCT04302454), which investigates the addition of ADT to metastasis‐directed radiotherapy vs metastasis‐directed radiotherapy alone in patients with limited PSMA‐PET/CT‐positive metastases in the bone and/or lymph nodes.^18^ In the recurrent M1a setting, after local therapy to the prostate, characteristics of the M1a LNs (number, location, and size), PSA kinetics, and time interval between primary treatment and diagnosis of M1a disease were considered the most important measures to decide on treatment by the experts. Based on a meta‐analysis, the main prognostic factors for oncological outcomes in patients treated for non‐metastatic PCa were short PSA doubling time (after RP) and short interval to biochemical failure (after radiotherapy).^33^ Also a high Gleason score was associated with worse survival outcomes.^33^ The EAU guidelines recommend integrating these factors to stratify patients with biochemical recurrence into low‐ and high‐risk categories.^18^ Additionally, in a retrospective, multicenter study including patients with a PSA rise and nodal recurrence following RP, three of more positive spots at PET/CT and retroperitoneal uptake at PET/CT were associated with early clinical recurrence following salvage LN dissection.^34^

About two‐thirds of the experts expressed the opinion that de novo M1a disease might be curative, especially when only one solitary LN is involved. Using more sensitive imaging techniques like PSMA‐PET/CT, one might select and treat patients with better prognostic factors. However, the evidence of potential survival benefit is currently lacking. There was no agreement on which endpoint should be used in clinical trials investigating the management of M1a PCa. In the absence of prospective randomized trials, the panel believed that a large national registry is needed to prospectively collect the data of patients treated for M1a PCa.

The absence of evidence from high‐quality clinical trials made the use of subjective opinions for many items around M1a management inevitable, which forms the most important limitation of this study. In conclusion, the panelists agreed that PSMA‐PET/CT is the preferred diagnostic modality to detect M1a PCa in both de novo and recurrent setting, but it is currently unknown if improvement in detection leads to better outcomes. Also, no consensus was obtained on the management of M1a PCa in daily practice. This may be a consequence of the scarcity of evidence from clinical studies and the lack of guidance for M1a PCa in the guidelines. In the absence of clinical prospective studies, a large national registry is needed to prospectively collect the data of patients treated for M1a disease.

## CONFLICT OF INTEREST

Dr Aluwini reports personal fees from Astellas, during the conduct of the study. Dr Oprea‐Lager reports other (institutional consultancy fee) from Astellas, during the conduct of the study; and an unrestricted grant from Janssen, outside the submitted work. Dr de Barros reports other (institutional research support) from Astellas, during the conduct of the study. Dr Mehra reports personal fees from Astellas, during the conduct of the study; grants and personal fees from Astellas, grants and personal fees from Janssen, personal fees from MSD, grants and personal fees from Pfizer, grants from Sanofi, personal fees from BMS, and personal fees from Bayer, outside the submitted work. Dr Stoevelaar reports other (institutional payment to Ismar Healthcare NV for supporting the study), during the conduct of the study. Dr van der Poel reports personal fees and non‐financial support from Astellas, during the conduct of the study.

## Supporting information

Supplementary Material

Supplementary Material
