# Supplementary material for: M1a prostate cancer: Results of a Dutch multidisciplinary consensus meeting
Source: BJUI Compass. 2021 Feb 3;2(3):159–68. doi: 10.1002/bco2.73 (PMC8988794; doi:10.1002/bco2.73)
Supplement: Supplementary file 1 — Supplementary Material [file BCO2-2-159-s002.docx]

**Supporting information S1.** Final survey following the video conference

**Starting points**

For this consensus study, the following patient population is considered:

- Patients with suspicious lymph nodes outside the pelvic region on imaging, without indications of other (bone/visceral) metastases

It may concern patients with:

- De novo M1a (primary diagnosis of prostate cancer)
- M1a as recurrent disease after primary local treatment

**DEFINITIONS**

- Following the TNM Classification and Dutch Guideline on Prostate Cancer, M1a is defined as distant metastases, solely consisting of non-regional lymph nodes.
- The regional lymph nodes are the nodes of the true pelvis, which are essentially the pelvic nodes below the bifurcation of the common iliac arteries. Laterality does not affect the N-classification.

**Question 1**

The TNM classification takes the iliac bifurcation as the anatomical lower limit for non-regional lymph node metastases. Which limit do you consider most relevant for therapeutic decision-making?

1. Iliac bifurcation
2. Aorta bifurcation
3. What is in line with the lymph node dissection template or irradiation field (dependent on therapeutic option)
4. Can’t judge

**Question 2 (statement)^[[1]](#footnote-1)^**

The following locations of lymph node metastases can be considered as M1a prostate cancer:

1. Inguinal
2. Pararectal

**DIAGNOSTIC EVALUATION**

Imaging

Diagnostic imaging for the detection of metastases in prostate cancer is mainly used in patients with intermediate or high risk of disease progression.

**Question 3**

Which of the following factors do you consider to be an indication for imaging with the purpose of metastatic screening (all types). [multiple answers allowed]

1. ISUP grade ≥2 (Gleason score 3+4)
2. ISUP grade ≥3 (Gleason score 4+3)
3. PSA >20 ng/mL
4. ≥cT2c
5. ≥cT3
6. Combination of lower staging factors than options a-e
7. Can’t judge

**Question 4**

Which imaging technique would you recommend as a standard for the initial evaluation of metastases in prostate cancer? [multiple answers allowed]

1. wbMRI
2. Conventional CT
3. Bone scan ± SPECT/CT
4. ^68^Ga- or ^18^F-radiolabeled-PSMA-PET/CT
5. ^18^F-NaF PET/CT
6. Can’t judge

**Question 5**

How appropriate (reliable/accurate) do you consider the following imaging techniques for the diagnosis of non-regional lymph nodes (M1a) in patients with de novo prostate cancer?

[very appropriate-appropriate-neutral-inappropriate-very inappropriate; can’t judge]

1. wbMRI
2. Conventional CT
3. Bone scan ± SPECT/CT
4. ^68^Ga- or ^18^F-radiolabeled-PSMA-PET/CT
5. ^18^F-NaF PET/CT

**Question 6 (statement)**

If M1a is suspected on CT scan, an extra PSMA-PET/CT scan should be performed if this may have therapeutic consequences.

[agreement scale: see question 2]

**Question 7 (statement)**

If a PSMA-PET/CT scan reveals inconclusive M1a disease, a targeted MRI should still be performed for confirmation.

[agreement scale: see question 2]

**Question 8 (statement)**

In most cases, imaging is sufficient to diagnose M1a disease and anatomopathological confirmation is not required.

[agreement scale: see question 2]

**Question 9**

Which of the following characteristics do you consider most relevant to evaluate a non-regional lymph node using conventional CT?

1. Size
2. Morphology
3. Location
4. Size and morphology
5. Size and location
6. Morphology and location
7. Size, morphology and location
8. Can’t judge

**Question 10**

For PSMA-PET/CT, which parameters, in addition to a higher uptake, do you consider relevant to evaluate a suspicious non-regional lymph node?

[multiple answers possible]

1. Size
2. Localisation
3. Substrate on CT
4. Can’t judge

**Question 11 (statement)**

In case of exclusive mediastinal/hilar lymph nodes, which are enlarged and show an increased uptake, it is unlikely these are metastases of prostate cancer.

[agreement scale: see question 2]

**Question 12 (statement)**

The presence of a supraclavicular lymph node, which shows increased uptake, may indicate a metastasis of prostate cancer, even if no other active lymph nodes are seen elsewhere. [agreement scale: see question 2]

Questions 13-15 apply to patients with M1a as recurrent disease after primary local treatment of prostate cancer.

**Question 13**

Which of the following do you consider the most important indication for imaging in patients with biochemical recurrence after radical prostatectomy?

1. PSA >0.2 ng/ml
2. PSA >0.5 ng/ml
3. PSA >1.0 ng/ml
4. Can’t judge

**Question 14**

Which of the following do you consider the most important indication for imaging in patients suspicious of recurrent disease after external beam radiation?

1. 3 consecutive PSA rises, independent of PSA level
2. PSA >2 above nadir (Phoenix criteria)
3. PSA >1.0 ng/ml
4. Can’t judge

**Question 15**

Which imaging technique do you consider appropriate for evaluation recurrent disease (M1a) after primary local treatment? [multiple answers allowed]

1. wbMRI
2. Conventional CT
3. Bone scan ± SPECT/CT
4. ^68^Ga- or ^18^F-radiolabeled-PSMA-PET/CT
5. ^18^F-NaF PET/CT
6. Can’t judge

**TREATMENT CHOICE**

We will distinguish between:

- Patient with de novo M1a (primary diagnosis of prostate cancer)
- Patients with M1a as recurrent disease after primary local treatment

**I. De novo patients**

**Question 16a**

Could treatment of de novo M1a prostate cancer have a curative intent?

1. No, never
2. In some cases
3. Often
4. Can’t judge

**Question 16b** (if question 16a was answered with a or b)

Which conditions are ‘potentially curative’? [multiple answers allowed]

1. Lymph nodes <1 cm
2. Lymph nodes <2 cm
3. <3 suspicious lymph nodes
4. <5 suspicious lymph nodes
5. Lymph nodes below the aortic bifurcation
6. Lymph nodes below the crossing of ureter and renal vessels
7. Lymph nodes below the diaphragma
8. Solitary lymph node

**Question 17**

What do you consider the most important endpoint in clinical studies into the treatment of M1a prostate cancer?

1. Overall survival
2. Metastasis progression-free survival
3. Delay of systemic treatment
4. Can’t judge

**Question 18**

Which of the following treatments could be an option for patients with de novo M1a prostate cancer?

[never/seldom, sometimes, often, can’t judge]

1. Radical prostatectomy
2. Radical prostatectomy + radiation or surgical treatment of M1a lesion(s)
3. Radical prostatectomy + ePLND
4. Radical prostatectomy + ePLND + radiation or surgical treatment of M1a lesion(s)
5. ADT alone
6. ADT + radiation therapy to the prostate
7. ADT + radiation therapy to the prostate and pelvic region
8. ADT + radiation therapy to the prostate + radiation therapy M1a lesion(s)
9. ADT + radiation therapy M1a lesion(s)
10. ADT + chemotherapy
11. ADT + new hormonal therapy (abiraterone, enzalutamide, apalutamide)

**Question 19**

What are the three most important parameters for treatment choice in patients with de novo M1a prostate cancer?

1. Stage primary tumour
2. Number of regional lymph nodes
3. Location (‘level’) of M1a lymph node(s)
4. Number of M1a lymph nodes
5. Size/intensity (uptake) M1a lymph node(s)
6. PSA value
7. Gleason score primary tumour
8. Can’t judge

**II. Patient with M1a as recurrent disease after primary local treatment**

Patient population

- Patients in whom M1a was diagnosed after primary treatment with curative intent (e.g. if imaging is performed after rising PSA)
- Exclusion: systemic treatment, unless as part of a treatment with curative intent

**Question 20**

Which of the following treatments could be an option for patients with M1a after primary treatment with curative intent?

[never/seldom, sometimes, often, can’t judge]

1. Deferred treatment
2. Local radiation of M1a lymph nodes
3. Local surgery of M1a lymph nodes
4. ADT alone
5. ADT + treatment M1a (local radiation/surgery)
6. ADT + chemotherapy/new hormonal therapies
7. Clinical study

**Question 21**

What are the 3 most important parameters for treatment choice in patients with M1a after primary local treatment?

1. Primary treatment of prostate cancer (RP, RT, RT+ADT)
2. Characteristics M1a lymph node(s) (location/number/size-intensity)
3. PSA kinetics
4. Time between primary treatment and diagnosis of M1a
5. Previous abdominal surgery
6. Previous abdominal radiation
7. Can’t judge

1. All statements to be rated on a 5-point agreement scale (strongly agree-agree-neutral-disagree-strongly disagree), with the additional option of “Can’t judge” [↑](#footnote-ref-1)
