# Supplementary material for: M1a prostate cancer: Results of a Dutch multidisciplinary consensus meeting
Source: BJUI Compass. 2021 Feb 3;2(3):159–68. doi: 10.1002/bco2.73 (PMC8988794; doi:10.1002/bco2.73)
Supplement: Supplementary file 2 — Supplementary Material [file BCO2-2-159-s001.doc]

**Supporting information S2**. Panel results on statements and questions on the management of M1a PCa

| **Statement [S]/question [Q]** | | **Panel outcome** |
| --- | --- | --- |
| **Definition** | | |
| 1. | [Q] The TNM classification takes the iliac bifurcation as the anatomical lower limit for non-regional lymph node metastases. Which limit do you consider most relevant for therapeutic decision-making? | No consensus nor fair agreement |
| 2. | [S] The following locations of lymph node metastases can be considered as M1a prostate cancer:   1. Inguinal 2. Pararectal | a. No consensus nor fair agreement  b. No consensus nor fair agreement |
| **Diagnostic evaluation** | | |
| 3. | [Q] Which of the following factors do you consider to be an indication for imaging with the purpose of metastatic screening (all types). | Consensus for ‘PSA >20 ng/ml’ and ‘ISUP grade ≥3’  Fair agreement for ‘≥cT3’ |
| 4 | [Q] Which imaging technique would you recommend as a standard for the initial evaluation of metastases in prostate cancer? | Consensus for ‘PSMA-PET/CT’ |
| 5 | [Q] How appropriate (reliable/accurate) do you consider the following imaging techniques for the diagnosis of non-regional lymph nodes (M1a) in patients with de novo prostate cancer? | Consensus for ‘PSMA-PET/CT’ |
| 6. | [S] If M1a is suspected on CT scan, an extra PSMA-PET/CT scan should be performed if this may have therapeutic consequences | Consensus |
| 7. | [S] If a PSMA-PET/CT scan reveals inconclusive M1a disease, a targeted MRI should still be performed for confirmation | Fair agreement that this is not valid |
| 8. | [S] In most cases, imaging is sufficient to diagnose M1a disease and anatomopathological confirmation is not required | Consensus |
| 9. | [Q] Which of the following characteristics do you consider most relevant to evaluate a non-regional lymph node using conventional CT? | Consensus for ‘size, morphology and location’ |
| 10. | [Q] For PSMA-PET/CT, which parameters, in addition to a higher uptake, do you consider relevant to evaluate a suspicious non-regional lymph node? | Consensus for ‘localisation’ and ‘substrate on CT’  Fair agreement for ‘size’ |
| 11. | [S] In case of exclusive mediastinal/hilar lymph nodes, which are enlarged and show an increased uptake, it is unlikely these are metastases of prostate cancer | Consensus |
| 12. | [S] The presence of a supraclavicular lymph node, which shows increased uptake, may indicate a metastasis of prostate cancer, even if no other active lymph nodes are seen elsewhere | Consensus |
| 13. | [Q] Which of the following do you consider the most important indication for imaging in patients with biochemical recurrence after radical prostatectomy? | Consensus for ‘PSA >0.2 ng/ml’ |
| 14. | [Q] Which of the following do you consider the most important indication for imaging in patients suspicious of recurrent disease after external beam radiation? | Fair agreement for ‘3 consecutive PSA rises, independent of PSA level’ |
| 15. | [Q] Which imaging technique do you consider appropriate for evaluation recurrent disease (M1a) after primary local treatment? | Consensus for ‘PSMA-PET/CT’ |
| **Treatment choice** | | |
| 16. | [Q] a. Could treatment of de novo M1a prostate cancer have a curative intent?  [Q] b. Which conditions are ‘potentially curative’? (if question 16a was answered with ‘In some cases’ or ‘Often’ | a. Fair agreement for ‘in some cases + often’  b. Fair agreement for ‘LNs below aortic bifurcation’ and ‘solitary LN’ |
| 17 | [Q] What do you consider the most important endpoint in clinical studies into the treatment of M1a prostate cancer? | Fair agreement for ‘metastasis progression-free survival’ |
| 18. | [Q] Which of the following treatments could be an option for patients with de novo M1a prostate cancer? | Fair agreement for ‘ADT + RT to the prostate (often)’ |
| 19 | [Q] What are the three most important parameters for treatment choice in patients with de novo M1a prostate cancer? | Consensus for ‘number of M1a lesions’ and ‘location (level) of M1a LNs’  Fair agreement for ‘number of regional LNs’ |
| 20. | [Q] Which of the following treatments could be an option for patients with M1a after primary treatment with curative intent? | No consensus nor fair agreement |
| 21. | [Q] What are the 3 most important parameters for treatment choice in patients with M1a after primary local treatment? | Consensus for ‘characteristics M1a LNs’ and ‘PSA kinetics’  Fair agreement for ‘time between primary treatment and diagnosis of M1a’ |

Consensus (strong agreement): ≥75% of the panellists chose the same option

Fair agreement: 50-74% of the panellists chose the same option

ADT = androgen deprivation therapy; CT = computed tomography; LN(s) = lymph node(s); ISUP = International Society of Urological Pathology; MRI = magnetic resonance imaging; PCa = prostate cancer; PSA = prostate-specific antigen; PSMA-PET/CT = prostate-specific membrane antigen positron emission tomography/computed tomography; [Q] = question; RT = radiotherapy; [S] = statement
